# Treprostinil for persistent pulmonary hypertension of the newborn, with early onset sepsis in preterm infant: 2 Case reports

**DOI:** 10.1097/MD.0000000000007303

**Published:** 2017-06-30

**Authors:** Bo Young Park, Sung-Hoon Chung

**Affiliations:** Department of Pediatrics, Kyung Hee University School of Medicine, Seoul, Korea.

**Keywords:** infant, nitric oxide, persistent pulmonary hypertension of the newborn, premature, treprostinil

## Abstract

**Rationale:**

Persistent pulmonary hypertension of the newborn (PPHN) is a syndrome of failed circulatory adaptation at birth with persisting increased pulmonary vascular resistance that is associated with high mortality rates especially in preterm infants.

**Patient concerns:**

We reported 2 cases of PPHN in preterm infants with respiratory distress syndrome and early onset sepsis refractory to therapy with vasopressors, inotropes, and inhaled nitric oxide (iNO), in whom treatment with treprostinil was successful.

**Diagnoses:**

Infants showed a difference of more than 10% between pre- and postductal saturation of peripheral oxygen by pulse oximetry. Echocardiogram showed flattened ventricular septum, right to left shunting through the patent ductus arteriosus, and tricuspid regurgitation velocity above 2.9 m/s.

**Interventions:**

The patients received treprostinil through central venous line because iNO therapy was not effective.

**Outcomes:**

Within 6 to 12 hours after treatment with treprostinil, the patients showed dramatic clinical improvement, and no systemic side effects were observed, including intraventricular hemorrhage (≥grade II).

**Lessons:**

IV treprostinil might be given to preterm infants with severe PPHN, who did not respond to conservative therapies, including iNO.

## 1 Introduction

Persistent pulmonary hypertension of the newborn (PPHN) is defined as a persistent elevation of pulmonary vascular resistance after birth, resulting in right-to-left shunting via the patent foramen ovale (PFO) or patent ductus arteriosus (PDA).^[1]^ PPHN is traditionally considered a condition of full-term and near-term infants, but is increasingly being diagnosed in preterm infants.^[2,3]^ PPHN occurs in 1.9 per 1000 live births and is associated with a mortality rate of about 10% in newborns; a marked risk of mortality has been found in preterm infants despite adequate medical therapy including inhaled nitric oxide (iNO), along with high-frequency ventilation, vasodilators, surfactant, and supportive measures including sedatives and inotropics.^[2,4–6]^

Treprostinil (Remodulin, United Therapeutics Corporation, Research Triangle Park, NC), a vasodilator usually used in adults with pulmonary arterial hypertension (PAH) (World Health Organization Group 1), is a synthetic analog of prostacyclin and was approved for PAH by the US Food and Drug Administration in 2002 for subcutaneous (SC) use, and in 2004 for intravenous (IV) use.^[7]^ Although treatment with treprostinil has improved pulmonary hemodynamics and functional capacity in adult patients with PAH,^[8]^ there are limited data on the acute treatment of PPHN with treprostinil, and there are no data for preterm infants. We report 2 cases of PPHN in preterm infants unresponsive to iNO, in whom treatment with treprostinil was successful.

### 1.1 Treprostinil sodium dosing

The optimal dosage and dose escalation of treprostinil for PPHN has not been established. The SC or IV route can be used; however, the drug was administered by a central IV line due to concerns about SC injection in preterm infants. Treprostinil was diluted with normal saline (0.1:99.9 mL), initiated at 5 ng/kg/min, and increased by 5 ng/kg/min every 60 to 120 minutes. Because of the life-threatening status, the dose was increased up to 20 ng/kg/min, depending on the effect of the drug.

## 2 Case report

**Figure 1:**
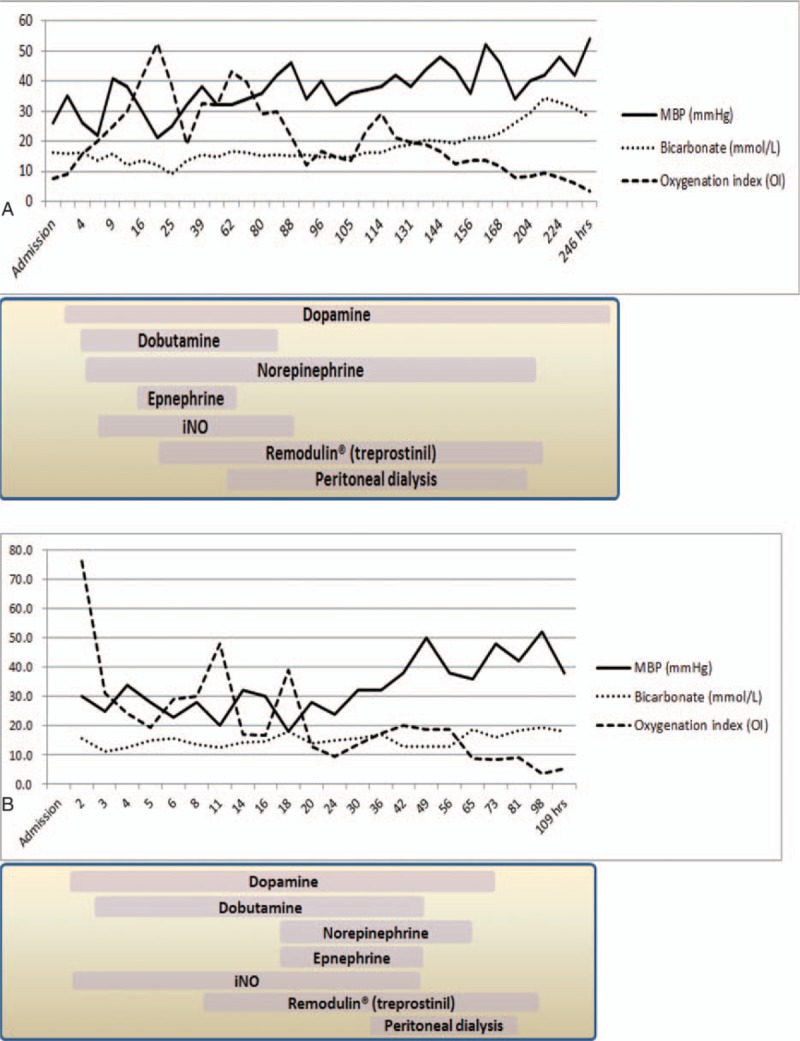
Mean arterial blood pressure, serum bicarbonate in blood gas analysis, and oxygenation index in relation to treprostinil therapy in the first (A) and second (B) patient. Horizontal axis indicates time (hours) since admission.

Maternal and neonatal characteristics and morbidities are summarized in Table 1, and brief clinical courses of the 2 cases are shown in Figure 1.

**Table 1:**
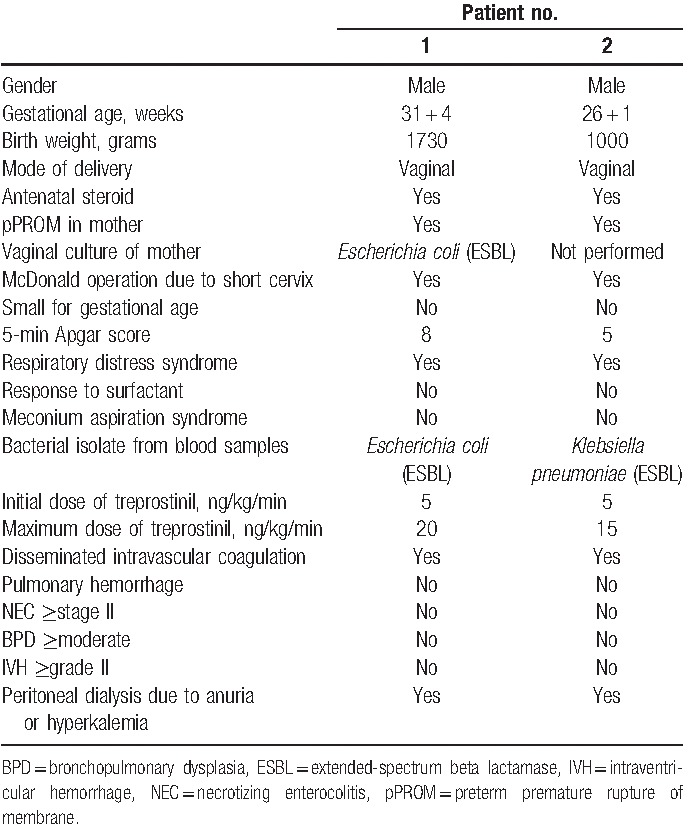
Patient characteristics.

### 2.1 Case 1

A preterm male infant was born weighing 1730 g to a 33-year-old G3P1 mother with preterm premature rupture of membrane and preterm labor at 31 and 4/7 weeks of gestation. At 17 weeks of postmenstrual age, the mother required cervical cerclage for an incompetent cervix. The Apgar scores were 7 and 8 at 1 and 5 minutes, respectively, but the infant required intubation and ventilation after moving to the neonatal intensive care unit (NICU) because he was hypotonic and showed central cyanosis. He was given artificial surfactant twice and maintained on a high-frequency ventilator with high settings. Initial laboratory findings included hemoglobin 17.3 g/dL, white blood cell (WBC) count 2710/μL, platelets 265,000/μL, C-reactive protein (CRP) 0.5 mg/dL (reference range, <0.5), arterial blood gases pH 7.007, pCO_2_ 65.3 mm Hg, pO_2_ 26.0 mm Hg, HCO_3_^−^ 16.4 mmol/L, and base excess (BE) –15.0 mmol/L, and serum glucose 113 mg/dL. Saturation of peripheral oxygen by pulse oximetry was 94% in the right arm and 49% in the right leg despite increasing FiO_2_ (fraction of inspired oxygen) and ventilatory support. Inotropics (dopamine and dobutamine), norepinephrine, epinephrine, and several saline boluses were administered to maintain a mean arterial blood pressure (MBP) above 30 mm Hg. The echocardiogram showed normal structural cardiac anatomy with flattened ventricular septum. Doppler showed right-to-left shunting through the PDA and PFO, and continuous-wave Doppler measurement of tricuspid regurgitation (TR) velocity of 3.5 m/s. iNO was started at oxygenation index (OI) 20.1 starting 7 hours after admission. Because maternal vaginal culture showed extended-spectrum β-lactamase (ESBL)-producing *Escherichia coli* colonization, ampicillin and meropenem were used as initial antibiotics for the treatment of probable neonatal early onset sepsis (EOS).

By day of life (DOL) 2, iNO was used for 12 hours and increased to 80 ppm; however, OI was increased to 52.5 without improvement and MBP dropped back to below 30 mm Hg. Treprostinil administration was initiated at 5 ng/kg/min starting 19 hours after admission and was increased up to 20 ng/kg/min by 3 hours due to lack of response and life-threatening status. BP gradually recovered to normal and OI began to decrease after increasing treprostinil to 20 ng/kg/min.

**Figure 2:**
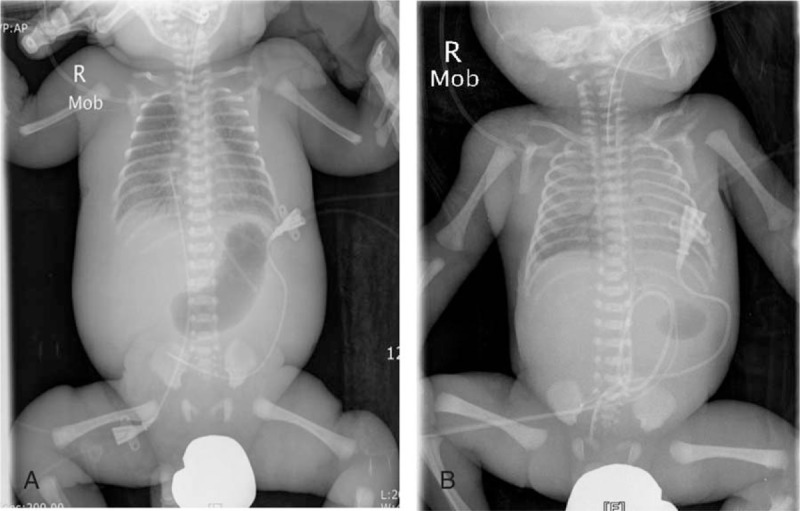
Inserted peritoneal dialysis catheter in the first (A) and second (B) patient.

The infant was anuric since DOL 1. By DOL 3, the infant's weight had risen to 2900 g, a 67.6% increase from birth weight, and the OI increased up to 43.1, with a decline in respiratory status. Laboratory findings revealed increased serum creatinine of 2.36 mg/dL and blood urea nitrogen of 64 mg/dL. A 4-F double-lumen central venous catheter (Arrow International, Reading, PA) was inserted at the bedside (Fig. 2A). Following catheter placement, manual peritoneal dialysis (PD) was initiated with 10 mL/kg volume dwells using 2.5% or 4.25% Hemosol BO (Gambro, Lundia AB, Sweden) for 24 cycles per day; there were no episodes of PD catheter insertion site leak. CRP increased to 8.6, but WBCs normalized to 6820/μL. Doppler showed left-to-right shunting through the 3.6-mm PDA and PFO, and continuous-wave Doppler measurement showed TR velocity of 2.4 m/s; we started weaning and discontinued iNO on DOL 4.

By DOL 5, ESBL-producing *E coli* grew on initial blood culture. Uremia improved with successful dialysis; the infant's weight began to decrease and serum creatinine decreased to 1.21 mg/dL; however, the infant required higher ventilator settings than the day before to maintain optimal blood gas levels due to congestive heart failure related to PDA, with B-type natriuretic peptide >5021 pg/mL. IV ibuprofen was used to treat PDA, and we confirmed the closed PDA on DOL 8. By DOL 7, urine output had improved during trials off PD. Norepinephrine, treprostinil, and PD were discontinued on DOL 8, and dopamine was discontinued on DOL 10. The infant was discharged to home on DOL 43, and both brain ultrasound examination and magnetic resonance imaging (MRI) performed before discharge were normal. He showed normal neurodevelopmental outcomes at 6 months of corrected age.

### 2.2 Case 2

The patient was a male neonate born at 26 and 1/7 weeks of gestation, weighing 1000 g. His mother required cervical cerclage due to a short cervix during pregnancy. His Apgar scores were 2 and 5 at 1 and 5 minutes, respectively. There was no response to stimulation at the time of birth, so he was intubated and given 1 dose of surfactant in the delivery room. The maternal CRP increased to 15.8 mg/dL immediately after delivery. Vital signs measured at the time of admission to the NICU: body temperature 36.4°C, heart rate 90 beats per min, oxygen saturation 48% in the right arm, and 29% in the lower limb; BP was not checked. Initial arterial blood gas analysis performed after inhaled oxygen concentration of 100% showed pH 6.705, PaCO_2_ 125.8 mm Hg, PaO_2_ 13.0 mm Hg, HCO_3_^−^ 15.7 mmol/L, and BE 20.0 mmol/L. WBCs 6980/μL and CRP 3.3 mg/dL were observed in the initial blood test. We used ampicillin and meropenem as initial antibiotics. Chest x-ray showed significant diffuse infiltrates in both lung fields, which was suspicious for neonatal respiratory distress syndrome (RDS). Echocardiogram demonstrated flattened ventricular septum and a right-to-left shunt through a 4.2-mm PDA, and grade 3 TR with velocity 3.9 m/s. After admission, surfactant was administered 2 more times, and high-frequency mechanical ventilation was started (FiO_2_ 1.0, mean airway pressure 18 mm Hg and stroke volume 50). The MBP was 20 to 25 mm Hg, and more than 20% difference between pre- and postductal saturation of peripheral oxygen by pulse oximetry persisted. We administered dopamine, dobutamine, norepinephrine, and epinephrine, and iNO was started at 20 ppm, but the difference in oxygen saturation was still significant and the blood pressure remained low. Then, treprostinil was started on the first DOL via the central venous line. The recommended dose was 2 ng/kg/min, but we started with 5 ng/kg/min due to life-threatening status and changed to 15 ng/kg/min after 3 hours. The initial response was observed and the oxygen saturation difference decreased to less than 10%.

The arterial blood gas analysis performed on DOL 2 showed pH 7.306, PaCO_2_ 30 mm Hg, PaO_2_ 47 mm Hg, HCO_3_^−^ 15.0 mmol/L, BE –11.0 mmol/L, and the MBP improved to 30 to 40 mm Hg, with a difference in oxygen saturation of less than 5%. Gram-negative rods were isolated on initial blood culture. The dose of iNO was gradually reduced and discontinued, and cardiovascular drugs other than dopamine were discontinued on DOL 3. Treprostinil was used at up to 15 ng/kg/min over 5 days, and the dose was decreased by 2 ng/kg/min at intervals of 8 hours by DOL 2. On DOL 5, ESBL-producing *Klebsiella pneumoniae* was isolated on initial blood culture. The echocardiogram showed that the direction of the right-to-left shunt was changed in both directions, the velocity of the TR improved to 1.4 m/s, and the size of the PDA was 3.3 to 3.5 mm. A 3-mm PDA was still present on DOL 27; IV ibuprofen was used for 3 days, and closure of the PDA was confirmed on DOL 30. By DOL 2, the patient received PD using a 4-F double-lumen central venous catheter (Arrow International) for 3 days due to acute renal failure with increased potassium above 7.0 mEq/L (Fig. 2B), and the symptoms improved and resolved. On DOL 16, the patient was extubated and maintained on nasal CPAP for a few more days. The vital signs finally stabilized without any respiratory assistance on DOL 49. Five brain ultrasound examinations were performed and the initial 4 examinations were normal. In the final examination, grade I intraventricular hemorrhage was observed on the right side, and brain MRI showed the same result. Due to retinopathy of prematurity, laser surgery was performed once, and he was discharged to home on DOL 83. He showed normal neurodevelopmental outcomes at 39 weeks of postmenstrual age, and did not need supplemental oxygen or other respiratory support.

### 2.3 Ethics statement

Data collection was approved by the institutional review board (IRB) of Kyung-Hee University Hospital at Gangdong. The informed consent requirements for this retrospective review were waived by the IRB (approved number, KHNMC NON2017-04-007).

## 3 Discussion

PPHN is associated with asphyxia, meconium aspiration syndrome, RDS, and sepsis/pneumonia.^[9]^ We present 2 cases of PPHN in preterm infants with RDS and EOS refractory to therapy with vasopressors, inotropes, and iNO, in whom treatment with treprostinil was successful.

RDS may be due to sepsis, and severe infection can cause constriction of lung vessels, resultant hypoxia may cause further pulmonary vasoconstriction and PPHN.^[10]^ Therefore, early detection and intervention is very important in PPHN. Specific interventions include administering oxygen, pulmonary vasodilators, and inotropes such as iNO, dopamine, dobutamine, epinephrine, and isoproterenol to decrease pulmonary blood pressure. Of these, iNO is considered to be the most effective method in PPHN; however, the use of iNO in preterm infants is controversial because of insufficient response and side effects such as intraventricular hemorrhage (IVH).^[11–13]^ In our cases, there was no IVH ≥grade II associated with the use of iNO, but there was no effect on PPHN. Several vasodilators have been used as additional therapy for PPHN, such as prostacyclin analogs (treprostinil, epoprostenol, and iloprost), endothelin-1 receptor antagonists (bosentan), and phosphodiesterase inhibitors (sildenafil). Among these, prostacyclin is an important mediator of pulmonary vasodilation, and deficiency is considered to be a major cause of vascular remodeling in PPHN.^[13]^

Treprostinil is a stable prostacyclin analog that is safe and easy to administer. It is given by SC or IV injection, and side effects include infusion site pain (SC), site infection, blood stream infection, flushing, diarrhea, nausea, jaw pain, and headaches in adults.^[7]^ However, in neonatal and pediatric patients, clinical studies are needed to confirm the safety and efficacy of the drug, and data on appropriate pediatric doses are not available. A few studies used an initial dose of 2 ng/kg/min and increased by 2 ng/kg/min every 8 to 24 hours in infants.^[14–16]^ However, we started at 5 ng/kg/min and increased by 5 ng/kg/min every 60 to 120 minutes, up to 20 ng/kg/min for 7 days in the first case, and up to 15 ng/kg/min for 5 days in the second case, because both were premature infants with RDS, PPHN, and EOS, which was life-threatening and unresponsive to iNO. Within 6 to 12 hours after treatment with treprostinil, both showed dramatic clinical improvement and no systemic side effects were observed, including IVH (≥grade II).

In our experience with 2 preterm infants who had PPHN-associated EOS, IV treprostinil was safe, efficacious, and well-tolerated. IV treprostinil might be given to preterm infants with severe PPHN, who did not respond to conservative therapies.
